# Supplementary material for: Splice-Junction-Based Mapping of Alternative Isoforms in the Human Proteome
Source: Cell Rep. Author manuscript; Available in PMC 2020 Jan 15. (PMC6961840; doi:10.1016/j.celrep.2019.11.026)
Supplement: 2 [file NIHMS1546469-supplement-2.zip › DF1/PXD010154/Esophagus/Esophagus_7_PCBP2_SGLDASAQTTSHELTIPNDLIGCIIGR.pdf]

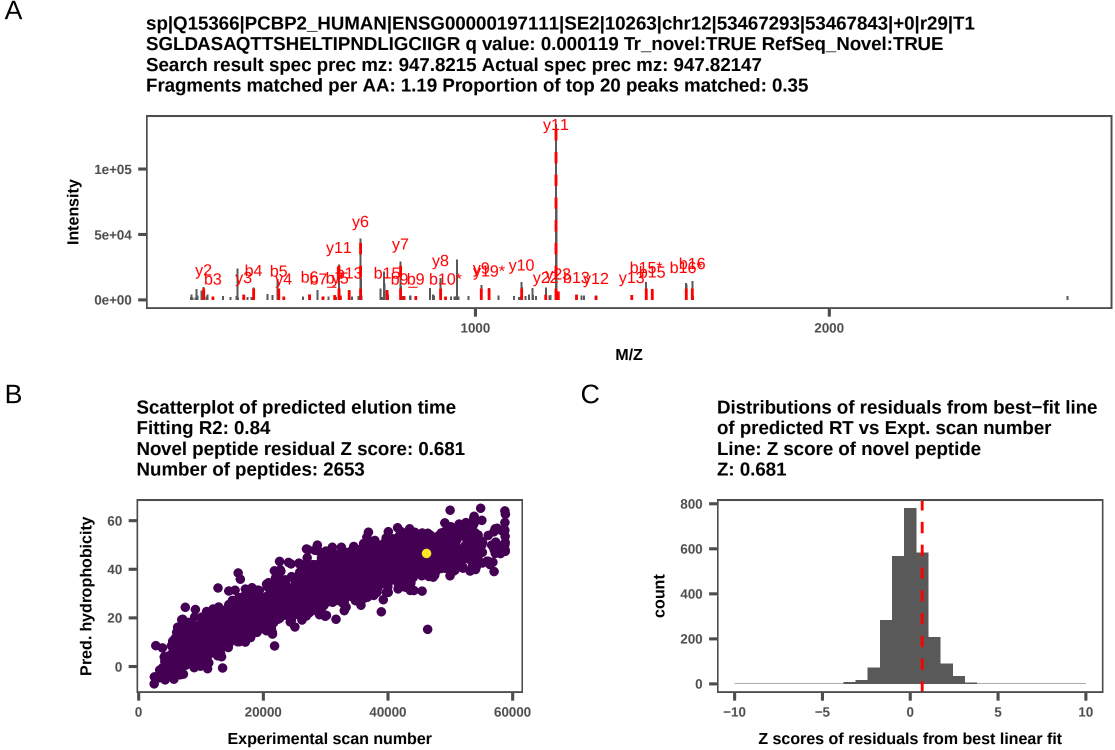
A
sp|Q15366|PCBP2_HUMAN|ENSG00000197111|SE2|10263|chr12|53467293|53467843|+0|r29|T1
SGLDASAQTTSHELTIPNDLIGCIIGR q value: 0.000119 Tr_novel:TRUE RefSeq_Novel:TRUE
Search result spec prec mz: 947.8215 Actual spec prec mz: 947.82147
Fragments matched per AA: 1.19 Proportion of top 20 peaks matched: 0.35
Intensity
1e+05
5e+04
0e+00
y11
y6
y11
y7
y8
y10
y12
y13
1000
2000
M/Z
B
Scatterplot of predicted elution time
Fitting R2: 0.84
Novel peptide residual Z score: 0.681
Number of peptides: 2653
Pred. hydrophobicity
60
40
20
0
0
20000
40000
60000
Experimental scan number
C
Distributions of residuals from best−fit line
of predicted RT vs Expt. scan number
Line: Z score of novel peptide
Z: 0.681
count
800
600
400
200
0
−10
−5
0
5
10
Z scores of residuals from best linear fit
